# Invasion, Distribution, Monitoring and Farmers Perception of Fall Armyworm (*Spodoptera frugiperda*) and Farm-Level Management Practices in Bangladesh

**DOI:** 10.3390/insects14040343

**Published:** 2023-03-31

**Authors:** Mohammad Shaef Ullah, Dilruba Sharmin, Toufica Ahmed Tumpa, Md Tafsir Nur Nabi Rashed, Powlomee Mondal, Md Wasim Akram, Setu Chowdhury, Masum Ahmad, Tetsuo Gotoh, Malvika Chaudhary

**Affiliations:** 1 Laboratory of Applied Entomology and Acarology, Department of Entomology, Bangladesh Agricultural University, Mymensingh 2202, Bangladesh; 2 National Pest Management Expert, Food and Agriculture Organization of the United Nations (FAO), Dhaka 1213, Bangladesh; 3 Faculty of Economics, Ryutsu Keizai University, Ryugasaki 301-8555, Ibaraki, Japan; 4 Asia Regional Coordinator—Plantwise, CABI, New Delhi 110012, India

**Keywords:** fall armyworm, transboundary pest, invasive species, farmers perception, integrated pest management, economic analysis of pesticide use

## Abstract

**Simple Summary:**

Fall armyworm (FAW), *Spodoptera frugiperda*, is a destructive agricultural pest due to its wide host range and adaptability to different climates. It was first detected in East Africa in 2016 and arrived in Bangladesh in 2018. The ideal climate in Bangladesh makes it easy for FAW to establish, infest, and cause outbreaks. Monitoring and scouting are crucial in controlling the pest. The management strategy of FAW involves both control methods and human action. However, there is a lack of research on human behavior and perception towards invasive pest management, which is important for understanding and predicting the success of disseminating information on pest management. Effective pest management requires a combination of accurate prediction of changes in the distribution and abundance of pests, and community engagement through the diffusion of information and technology. Strengthening global collaboration to improve biosecurity defenses is crucial to prevent transboundary insect pest invasions and protect food security, biodiversity, and human health.

**Abstract:**

Fall armyworm (FAW), *Spodoptera frugiperda*, is a major pest of maize that was first detected in Bangladesh in 2018 and rapidly spread throughout the maize-growing areas. The presence of FAW was monitored using sex pheromone traps. Farmers’ pest management practices were assessed through a questionnaire. The damage is most apparent in the early and late whorl stages. As the crop is grown mostly from November to April, both vegetative and reproductive growth stages remain vulnerable to extensive damage. The survey results showed that 100% of the farmers used pesticides for FAW control, 40.4% handpicked and crushed egg masses, 75.8% handpicked and crushed caterpillars, and only 5.4% used other techniques like applying ash/sand in the funnel of maize. Commonly used pesticides included Spinosad, Emamectin benzoate, Imidacloprid, and others. Thirty-four percent of farmers applied pesticides twice in a season and 48% applied pesticides three times in a season and 54% and 39% of farmers sprayed chemicals at 7-day and 15-day intervals, respectively. FAW causes an average economic loss of 37.7% in maize production without pesticides. Increased use of pesticides to control FAW poses hazards to human health, wildlife, and the environment, and is expensive. Therefore, well-tested agroecological practices and bio-control agents are needed for sustainable FAW management.

## 1. Introduction

Fall armyworm (FAW) *Spodoptera frugiperda* (J. E. Smith) (Lepidoptera: Noctuidae), in which *frugiperda* is Latin for “lost fruit”, is a native species of tropical and sub-tropical regions of the Americas [1]. It has a wide host range of over 353 plants belonging to 76 families, mainly from Poaceae (106), Asteraceae (31), and Fabaceae (31), although it has a preference for maize and rice [2,3]. FAW has two distinct strains: the corn-strain (C-strain) and rice-strain (R-strain), which have morphological similarities but differ in host range [4], mating behaviors [5], genetics [2] and pheromone components [4]. The C-strain primarily feeds on maize, cotton, and sorghum, while the R-strain feeds on rice and pasture grasses [2]. However, there have been cases of one strain being found in habitats dominated by the other strain [6]. The more prevalent strain among the two is the C-strain, which feeds on maize leaves and stems.

FAW appears to have invaded the Eastern Hemisphere about 8 years ago. The pest was able to travel approximately 7000 km from Nigeria to India between 2016 and 2018, and another 4000 km to Southeast Asia in the following year. Wind patterns and a readily available corn supply along its migration route in North America have enabled FAW to migrate thousands of kilometers annually. The spread of FAW in the Eastern Hemisphere, starting from a western African entry point in 2016, was largely facilitated by human transportation and trade. It is believed that FAW arrived in Asia through international trade and established in a suitable environment. A tropical and warm climate with temperatures ranging from 17–35 °C and mean annual rainfall ranging from 0–400 mm is considered ideal for the growth and survival of FAW [7].

**Figure 1:**
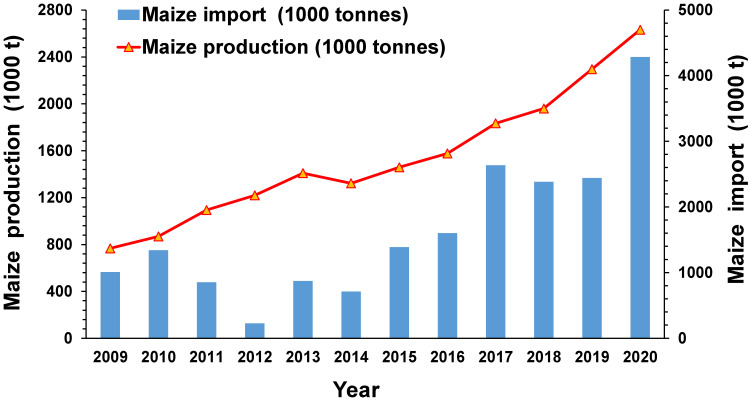
Rapid growth of maize production and imports in Bangladesh from 2009 to 2020 (Source: https://knoema.com/ (accessed on 11 January 2023)).

FAW infestations can cause yield losses of maize ranging from 15–73% [8]. The economic losses caused by FAW in Ghana and Zambia have reached USD 177.3 million and USD 159.3 million, respectively [9]. In China, the estimated financial loss of maize caused by FAW ranges from USD 5.4 to 47 billion per year [10]. Bangladesh is particularly concerned about the invasion of FAW, as maize has become the second most widely grown cereal crop in the country, second only to rice. The majority of maize produced in Bangladesh is sold on the market and can earn farmers over USD 1000 per hectare. In Bangladesh, maize production lags maize imports (Figure 1); the country hopes to cease maize imports by 2030. Currently, about 74% of maize is grown domestically due to the expansion of the poultry feed industry in Bangladesh, while the remaining 26% is imported at an annual cost of USD 0.4 billion to meet demand [11].

**Figure 2:**
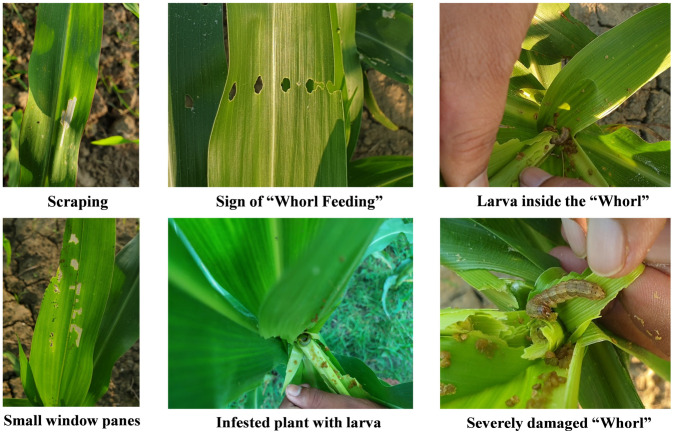
Different damage symptoms in maize caused by fall armyworm larvae.

Maize production in Bangladesh has faced several challenges in recent years, leading to decreased productivity and yields. Abiotic and biotic factors such as disease, drought, and now the major one, FAW, lower productivity of maize in Bangladesh [12,13]. FAW causes more damage at the larval stage as compared to other species. Young caterpillars feed superficially, and feeding is more active during the night. The matured larvae in the whorls of the plant feed on maize cob or kernels which reduces yield and quality (Figure 2).

Given FAW’s ability to impact staple and economic crops globally and locally, there is a pressing need for information on its potential distribution and environmental limitations. This information is critical for conducting regional and national pest risk assessments and devising effective management strategies. Early detection of infestations is crucial, as chemical insecticides are most effective when the larvae are small [14]. Effective pest management practices can help reduce crop losses, but largely depend on farmers’ knowledge, attitude, and behavior towards pest management. Therefore, it is important to understand how much farmers know about insect pests, yield damage, and effective management practices through surveys. However, current farmer practices for pest management lack a solid scientific foundation and their reasons for using these methods are unreliable. The aims of the current study were to provide a comprehensive understanding of the invasion, distribution, monitoring, and management practices of FAW in Bangladesh. The scope of the paper covers its abundance and distribution over time in Bangladesh, as well as the perception of farmers regarding FAW. The paper also aims to understand what farmers know about insect pests, their perceptions about crop yield damage, the control methods they choose to apply, and the perceived effectiveness of these methods, and to propose alternative options for smallholder farmers. Additionally, the paper highlights the economic impact of FAW infestation on maize production in Bangladesh and suggests the need for a coordinated global response to manage FAW invasion and limit its spread.

## 2. Materials and Methods

### 2.1. Investigation of Invasion and Distribution of Fall Armyworm in Bangladesh

**Figure 3:**
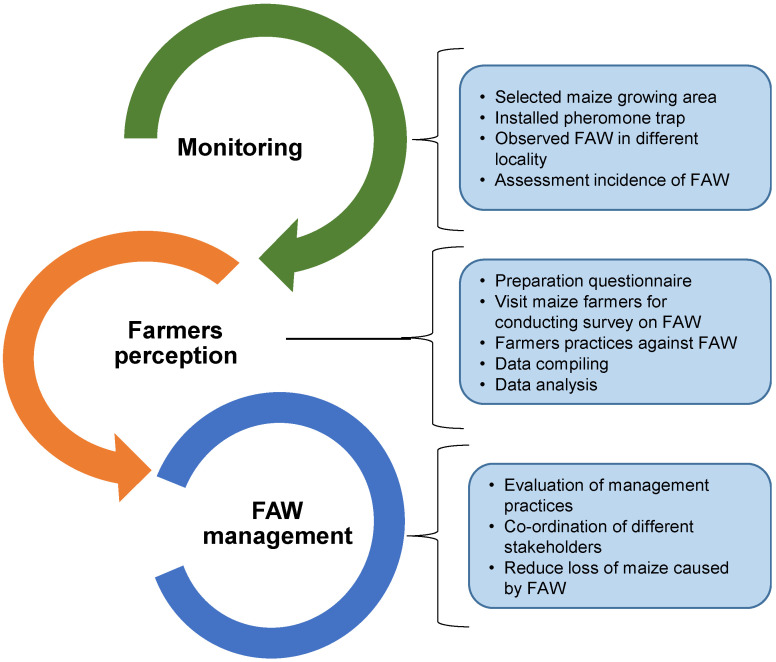
Outline of activities from monitoring to management of FAW.

The likely distribution of FAW in Bangladesh was compiled using multiple sources: (1) the Department of Agriculture Extension (DAE), Ministry of Agriculture, Government of Bangladesh, (2) installation of sex pheromone traps in different districts and monitoring their presence, and (3) consultations between DAE officials at the sub-district and district levels and local experts in various locations, using trap catch recorded data from different districts of Bangladesh. Monitoring of FAW through trapping involves regular observations and recording of FAW infestation and damage in the field, while farmers survey data refers to the collection of information on FAW distribution, prevalence, and impact across a larger geographical area. The two are connected in that monitoring data can be used to inform and validate survey data and survey data can be used to identify areas that require increased monitoring efforts. By combining both types of data, researchers and policymakers can gain a better understanding of the spread and impact of FAW, as well as identify effective management strategies (Figure 3).

### 2.2. Monitoring of Fall Armyworm in Bangladesh

**Figure 4:**
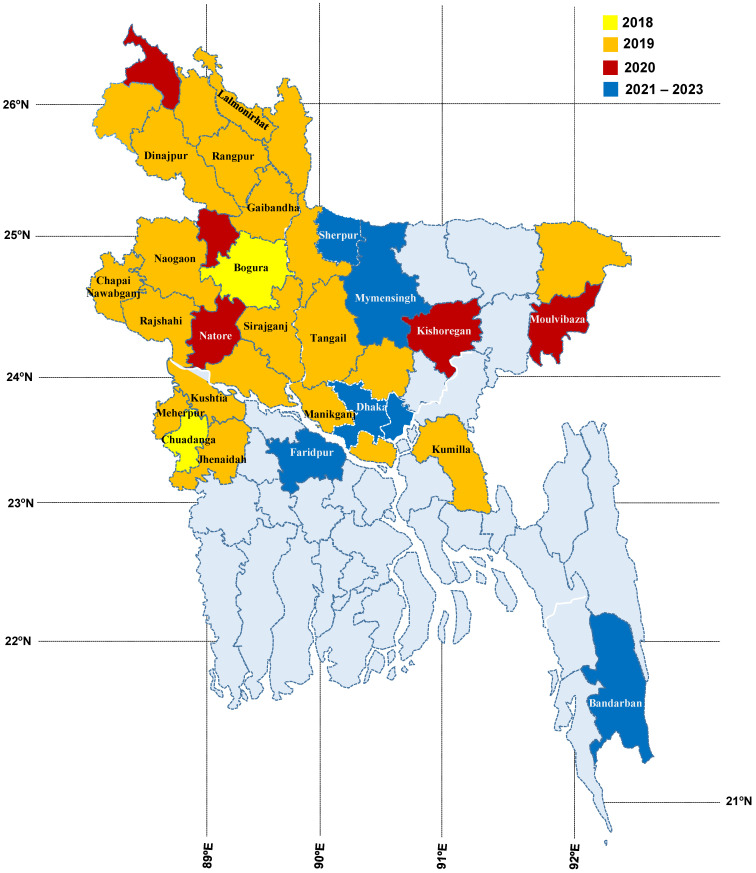
Distribution of fall armyworm from 2018 to 2023 in Bangladesh (different shades indicate the invasion in areas reported in the years). Districts mentioned in the map are where monitoring of fall armyworm was conducted using pheromone traps.

**Figure 5:**
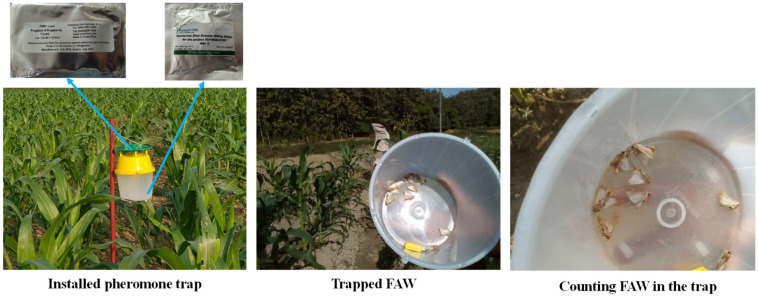
Pheromone traps for monitoring fall armyworm in a maize field.

Twenty-six major maize growing districts in Bangladesh were selected for FAW monitoring (Figure 4). Five traps were installed in each location for monitoring purposes. A commercial lure produced by Chemtica International, Costa Rica (Frugilure S-*frugiperda*) was placed inside a bucket-type trap and positioned in the center of the field shortly after plant emergence. A slow-releasing killing strip (Russell IPM) was placed inside the trap and secured to a wooden stem at an initial height of 1 m above the soil surface. As the plants grew, the trap was lifted upward to ensure it remained above the plants. Traps were installed in every hectare in each location and the lure and killing strip were replaced every eight weeks. The number of insects captured in the traps was recorded weekly (Figure 5) by observing the number of male moths caught on each observation date and emptying the traps.

### 2.3. Farmer Perception and Farm-Level Management Practices of Fall Armyworm

The study on farmer perception and farm-level management practices of FAW was conducted in ten major maize-growing districts in Bangladesh, representing diverse farming systems and livelihood strategies. Ten districts from these areas were selected purposively, targeting those with a known occurrence of FAW from former surveys and reports of government extension personnel (Table 1). A representative sample of 223 households was selected through purposive sampling and interviewed from November 2020 to June 2021 using a semi-structured questionnaire administered by trained enumerators in the local language. The head of the household, responsible for farm decisions, was selected as the respondent. The questionnaire evaluated farmers’ pest management decisions based on their knowledge, perceptions, and practices and focused on: (1) basic information of the interviewee, such as gender and cultivable land owned; (2) practices for maize production, such as varieties grown and experience; (3) experience with FAW infestation, such as damage season and month of highest infestation; and (4) farmer’s knowledge, perceptions, and practices concerning FAW management. Farmers’ control measures for FAW were also considered, and additional information was obtained on pesticide selection and usage for those who used pesticides (File S1).

### 2.4. Economic Benefit of Pesticide Use

The maize yield loss was determined as the difference between attainable yield and actual yield with and without the use of pesticides to manage FAW. The data was obtained from farmers’ perceptions. The process involved asking farmers to first calculate their actual maize yield in the community without using pesticides, taking into account all production constraints including FAW. Next, they were asked to estimate the attainable yield if they had used pesticides and considered all production constraints. The yield gap was calculated with and without pesticide use, and this information was used to determine the benefit of pesticide use for managing FAW.

### 2.5. Statistical Analyses

Pheromone trap catch data was normalized prior to analysis. Assumptions were conducted to ensure the data met the requirements before conducting the ANOVA. The number of moths caught per trap per week in the experiment was analyzed through a one-way analysis of variance (ANOVA) to identify variations in FAW infestation at different times in the study area using SPSS version 26 (IBM SPSS Statistics for Windows, Version 26.0. Armonk, NY: IBM Corp). Raw survey data was coded and categorized, and necessary analyses were conducted using SPSS version 26. Farmer perceptions were summarized through cross-tabulations and described using means and percentages. Chi-square analysis was performed to compare the responses.

## 3. Results

### 3.1. Invasion and Distribution of FAW in Bangladesh 

FAW is a migratory species with a wide dispersal range and was first identified in Bangladesh in November 2018 on maize in the Bogura and Chuadanga districts. Within a short period of time, FAW rapidly spread throughout Bangladesh’s major maize growing areas, posing a significant threat to maize production. It then spread to Bangladesh in late 2018, where it was detected in 24 districts by May 2019, primarily in areas where maize is a significant crop. As time progressed, FAW has spread to all regions where maize is grown and has now been observed in 35 major maize-growing districts in Bangladesh (Figure 4).

### 3.2. Monitoring of FAW in Bangladesh

**Figure 6:**
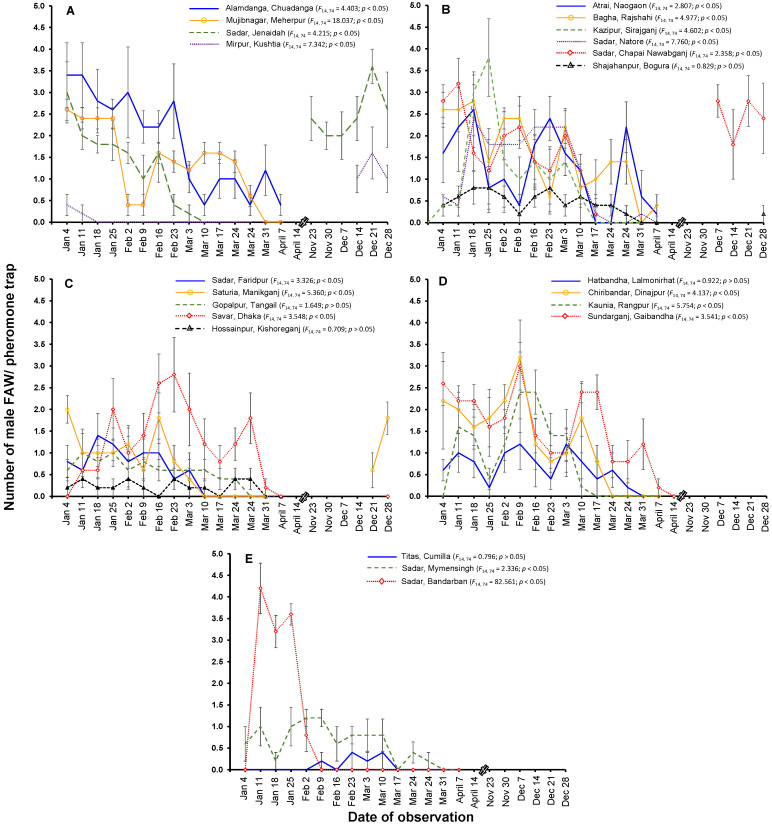
Numbers of fall armyworm adult male catches in pheromone traps in different districts in Bangladesh in maize fields. (**A**) Khulna division; (**B**) Rajshahi division; (**C**) Dhaka division; (**D**) Rangpur division; (**E**) Chattagram and Mymensingh divisions. Data are shown as mean ± SE. No adult FAW was trapped in two districts (Sherpur and Moulvibazar).

The results of the pheromone trap catches revealed significant variations in insect abundance across the different dates of observation (Figure 6). During the 2020–2021 maize season, FAW was detected in 24 districts throughout Bangladesh, although no adult FAW were captured in the pheromone traps in the Sherpur and Moulvibazar districts. The number of catches of male moths using pheromone traps in different districts varied significantly with time (*p* < 0.05). The FAW moths were captured more in the northwestern and southwestern regions in Bangladesh. FAW infestation started earlier in the southwestern region, where maize is cultivated earlier than in other regions of Bangladesh. The highest frequency of adult FAW was observed through trapped catch in the early stages of maize growth (early and late whorl stages), rather than in the later stages of tasseling and cob formation. At this stage, maize seedlings are vulnerable to damage from larvae and can be killed by the pests. The variation in the number of moths caught per trap in different districts in Bangladesh reflects the population fluctuations (Figure 6).

### 3.3. Farmer Perception and Farm-Level Management of FAW 

Maize is now mainly grown in the northwestern, southwestern and central regions of Bangladesh. The difference of maize productivity among regions is affected by some natural phenomena, such as rainfall, temperature, humidity and other agricultural ecological characteristics, which are relatively unfavorable to backward regions. All the farmers interviewed reported FAW infestations, with a majority indicating that it occurred on their own farms.

#### 3.3.1. Maize Production Systems

The majority of the respondents (98.7%) were male and directly involved in maize cultivation. Most maize production in the study area was small-scale, with 86.6% of farmers cultivating less than three acres of land. This accounted for a significant portion of the total cultivated land used by households (Table 2). The majority of farmers (36.8%) had been engaged in maize cultivation for more than six years and most of the farmers grow hybrid maize varieties that they also perceived to be more susceptible to FAW than local varieties. (Table 2).

#### 3.3.2. FAW Infestation Knowledge

FAW primarily infested maize plants from mid-November to mid-March. The infestation started at the seedling stage, but the early and late whorl stages were most affected. The infestation of FAW in different crop stages was significant and varied with the farmer’s maize cultivation experience (χ^2^ = 13.74; *p* < 0.05). The prevalence of FAW infestation based on maize plant damage was highest in January, February, March, and April compared to other months (χ^2^ = 50.51; *p* < 0.05) (Table 3).

#### 3.3.3. Farmers’ Knowledge, Perceptions, and Practices concerning FAW Management

All farmers used at least one method to manage FAW during the maize growing season (Table 3). A total of 100% of the farmers used pesticides for FAW control, 40.4% handpicked and crushed egg masses, 75.8% handpicked and crushed caterpillars, and low participants used other techniques like applying ash (5.4%)/sand (2.7%) in the funnel of maize (Table 3). Biocontrol agents, including farm-based or home-based plant extracts such as chili pepper, neem, and tobacco, were used by only 1% of the farmers. 

Pesticides were normally applied during the seedling and vegetative growth stages of maize. FAW was detected earlier in the monitoring (November–December) in several areas, indicating that they infested the maize plants at the seedling and vegetative stages. The number of pesticide applications during the growing season was significantly affected by the farmers’ growing experience of maize (ꭕ^2^ = 24.13, *p* < 0.05). Over 33.6% and 47.5% of farmers applied pesticides twice or thrice during the maize growing season, and more than 53.8% and 38.6% of farmers sprayed chemicals at 7-day and 15-day intervals, respectively, to manage FAW larvae. The interval between pesticide applications was also significantly affected by the farmers’ maize growing experience (ꭕ^2^ = 29.02, *p* < 0.05). Farmers estimated that the damage caused by FAW would be more than 40% yield loss if they did not take any control action (Table 3).

#### 3.3.4. Pesticides Used by Farmers

During the survey period, 26 pesticides were utilized to control FAW. The most frequently used pesticides were Chlorpyrifos (50%) and Cypermethrin (5%) in a mixture, followed by Spinosad, Lambda Cyhalothrin, Emamectin Benzoate, and a mixture of Thiamethoxam (20%) and Chlorantraniliprole (20%) (Table 4). According to the World Health Organization (WHO), Chlorpyrifos, Cypermethrin, Thiamethoxam, Emamectin Benzoate, and Lambda Cyhalothrin are classified as Class II (moderately hazardous) pesticides, while Spinosad is classified as Class III (slightly hazardous) (Table 4).

#### 3.3.5. Economic Benefit of Pesticide Use

The average yield of maize during the study period was 11 metric tons per hectare, which is equivalent to 25 maunds per hectare in Bangladesh when pesticides were used to manage FAW. On average, the loss of maize yield without pesticides was 4.15 metric tons per hectare, which represents a 37.7% decrease. Based on the current market price in Bangladesh, it was estimated that the attainable yield of maize was 330,000 BDT (approximately USD 3220) while the actual yield without pesticides was 205,500 BDT (approximately USD 2005).

## 4. Discussion

The spread of invasive pests and diseases is affecting global agriculture, with international trade often playing a role in their expansion. FAW, originally from the Americas, has now infiltrated over 70 countries [15,16,17,18,19,20,21,22,23]. However, Europe’s Mediterranean region still poses a risk of future invasion. The monitoring of FAW data and farmers’ survey data of FAW are connected in that they both provide important information about the presence and impact of FAW in a given area. Monitoring of FAW on a regular basis helps us to detect and monitor the presence and abundance of FAW in different areas in Bangladesh. Distribution and abundance maps of FAW can be used to identify high-risk areas and prioritize interventions. This information helps in planning interventions such as targeted spraying, use of biocontrol agents, and deployment of resistant maize varieties. Concentrating efforts in the northwest, southwestern and central districts where maize is mainly grown in Bangladesh is a good idea. Understanding the perceptions of farmers on FAW damage and management is important to develop effective and sustainable control strategies. Farmer participatory approaches such as farmer field schools and community-based monitoring can be used to gather this information. This data can be used to design effective extension messages, develop integrated pest management strategies, and improve the adoption of control measures. By using a combination of these strategies, it is possible to effectively monitor and manage FAW infestations, reduce crop losses, and promote food security. 

The rapid spread of FAW in Bangladesh’s cropping season is largely due to its exceptional flight ability. The pest can migrate great distances on wind currents and breed continuously in areas with suitable climate. Bangladesh’s climate is conducive to FAW establishment, infestation, and outbreak [7]. Its long border with India may also contribute to pest dispersion in the region. 

Effective monitoring and scouting are essential for timely responses to the presence and growth of pests, particularly cross-border invasive insects that can harm crop health. These efforts should be financially viable and aimed at keeping the FAW population below the economic threshold level. An important first step in understanding the extent of a species’ invasiveness is accurately predicting its distribution and abundance over time and space [24]. FAW moths migrate to maize fields soon after plant emergence, as shown by the sex pheromone trap data. The low number of FAW males caught in some districts (Figure 6) might indicate that the males in the study area are less responsive to commercial sex pheromone lures than those in other areas. However, this low population could also result from chemical insecticides applied in adjacent areas. The high natural enemy population in the area may also play a role, but this was not investigated. The infestation level of FAW in Bangladesh was more severe during the 2020–2021 winter season compared to other seasons. This is likely due to the warmer temperatures in some locations and the lack of late-season rainfall. Late-season heavy rainfall can physically remove FAW larvae from plants or drown them in maize whorls, but this season has seen less rainfall than other seasons, which may have contributed to the higher rates of attack seen so far.

Effective pest management of an invasive species such as FAW depends on both control methods and human actions. While control methods are continuously being refined, not enough research is being devoted to the human factor in invasive pest species management. However, the wide diversity in community behaviors and perceptions toward new information and technology regarding invasive pest management (i.e., behavioral heterogeneity) is critical for understanding and predicting the success of disseminating information on pest management throughout the community. It is also reported that maize varieties have fluctuating susceptibility, with hybrids and open pollinated varieties were more vulnerable to FAW damage at early growth stages, but they grew out of it through the mid to late whorl stages [25]. Pesticide application was the most frequently used approach for FAW management, with a prevalent habit for farmers not to follow safety precautions. The invasion of FAW in Bangladesh in late 2018 created a great deal of fear and desire to protect the crops. Therefore, it is not surprising that farmers tend to apply pesticides as the primary control method for FAW. Although different development agencies provide support with pheromone traps, lures, and killing strips, they are used only for monitoring purposes.

Frequent use of chemical pesticides can have negative effects on the environment, human, and animal health. Overuse can also lead to the development of resistance, making control difficult [26]. In Bangladesh, many farmers apply pesticides two or three times per season, which may contribute to increased resistance. Non-chemical alternatives, such as ash/soil and plant extracts, have also proven effective in controlling FAW and offer low-cost options for small-scale farmers. Ash is used as a repellent as an age-old practice. This is very conventional and used frequently. Sand is also effective against caterpillars because its roughness kills or repels them. These alternatives pose low risks to health and the environment, making their inclusion in FAW management research crucial. Understanding farmers’ perceptions of these alternative methods and their effectiveness in controlling FAW could lead to their wider adoption once they are validated in the field. Attitudes and perceptions towards risk among farmers are crucial factors that impact farming practices, investment decisions, and the implementation of diverse risk management strategies [27]. Other cultural practices, such as regular weeding, intercropping, and trap cropping, can also reduce FAW infestation [28]. Intercropping systems, such as push–pull technology and maize intercropped with leguminous crops, have been shown to offer better protection from FAW and other complex pests compared to monocropped maize [29].

FAW is causing significant economic and food security losses in Ethiopia, where it causes an average annual loss of 36% in maize production and destroyed 0.67 million tons of maize between 2017 and 2019, equating to USD 200 million or 0.08% of the gross domestic product [30]. Similar losses are seen in other countries, including Ghana and Zambia (22–67%) [31], Kenya (47%) [32], and Zimbabwe (9.4%) [33]. These losses pose a significant threat to the livelihoods of many poor people who depend on maize production. Additionally, the frequent use of chemical pesticides to control FAW threaten sustainable food production in Bangladesh. The data from this study are limited in that they do not consider the timing of control measures or the stage of the insects’ growth, and are not nationally representative. Further studies are needed to address these limitations and to effectively allocate resources for controlling FAW. In addition, FAW prefers warm temperatures and high humidity, which are typical of tropical and subtropical regions. In Bangladesh, FAW infestations have been reported in different parts of the country, but the severity of the infestations may vary depending on the latitude and climatic conditions of each region [34]. For example, FAW infestations may be more severe in the southwestern and northwestern parts of Bangladesh, which are closer to the equator and have a more tropical climate. However, other factors such as crop management practices and the availability of natural enemies can also influence the incidence and severity of FAW infestations in different regions. 

The highly mobile and genetically diverse FAW presents many technical difficulties for research on its migration and population dynamics [35]. To address these issues, a unified global response is needed and is beginning to emerge. Organizations like the Ministry of Agriculture (Bangladesh), CIMMYT, FAO, CABI, and USAID are coordinating efforts against FAW in Bangladesh and providing support for early warning tools, farmer field schools for integrated pest management, and a food security risk assessment model. Effective and low-toxicity products, such as the biopesticide Fawligen and the seed treatment Fortenza, have been quickly registered for use against FAW. Identifying native natural enemies (predators, parasitoids, microbials etc.) of FAW and developing effective biological control strategies that are compatible with local agronomic practices is a top priority. A National Task Force and R&D consortium (Bangladesh) have been established to monitor and develop integrated pest management solutions that include host plant resistance, safer chemical pesticides, biological and cultural control methods, and agronomic management. To protect food security, biodiversity, and human health, it is crucial to enhance biosecurity defenses and strengthen global collaboration to prevent the spread of transboundary insect pests.

## Figures and Tables

**Table 1:** Study locations and sample sizes for farmers’ perception study.

Agro-Ecological Zone (AEZ)	Division	District	Upazila	No. of Respondent Farmer
Zone I—Old Himalayan Piedmont Plain	Rangpur	Dinajpur	Birol	20
Zone 3—Tista Meander Floodplain	Rangpur	Rangpur	Badarganj	31
	Rangpur	Lalmonirhat	Aditmari	11
	Rangpur	Kurigram	Rajarhat	23
	Rangpur	Gaibandha	Palashbari	25
Zone 7—Active Brahmaputra—Jamuna Floodplain	Dhaka	Dhaka	Dhamrai	16
Zone 9—Old Brahmaputra Floodplain	Mymensingh	Sherpur	Nalitabari	17
AEZ 10—Active Ganges Floodplain	Khulna	Kushtia	Bheramara	39
Zone 11—High Ganges River Floodplain	Khulna	Chuadanga	Sadar	20
	Rajshahi	Chapai Nawabganj	Nachole	21

**Table 2:** Demographics of maize farmers in this study.

Farmer Category		Frequency (Number)	Percentage (%)
Sex			
	Male	220	98.7
	Female	3	1.3
Land owned for maize cultivation (acre)			
	<1	74	33.2
	1	77	34.5
	2	42	18.8
	3	14	6.3
	4	11	4.9
	>4	5	2.3
Experience in maize farming (years)			
	0–3	67	30.0
	4–6	74	33.2
	>6	82	36.8
Maize variety cultivated			
	Local	0	0.00
	Hybrid	223	100.00

**Table 3:** Perceptions of maize farmers of fall armyworm infestations and management.

Parameters ^a^		Experience (in Years) of Cultivating Maize			
		0–3	4–6	>6	Total
FAW infested cropping stage *					
	Early whorl stage	1 (0.5)	4 (1.8)	6 (2.7)	11 (4.9)
	Late whorl stage	57 (25.6)	65 (29.2)	75 (33.6)	197 (88.3)
	Tasseling/Silking	3 (1.4)	4 (1.8)	1 (0.5)	8 (3.6)
	Mature/Cob formation	6 (2.7)	1 (0.5)	0 (0.0)	7 (3.1)
FAW infested more in the month ***					
	January	10 (4.5)	10 (4.5)	15 (6.7)	35 (15.7)
	Feb	29 (13.0)	24 (10.8)	20 (9.0)	73 (32.7)
	March	13 (5.8)	8 (3.6)	9 (4.0)	30 (13.5)
	April	5 (2.2)	23 (10.3)	9 (4.0)	37 (16.6)
	May	7 (3.1)	1 (0.5)	1 (0.5)	9 (4.0)
	June	0	0	0	0
	July	0	0	0	0
	August	0	0	0	0
	September	0	0	0	0
	October	0	3 (1.3)	7 (3.1)	10 (4.5)
	November	1 (0.5)	5 (2.2)	12 (5.4)	18 (8.1)
	December	2 (0.9)	0	9 (5.4)	11 (4.9)
Methods used to control FAW					
	Chemical	2 (0.9)	4 (1.8)	1 (0.5)	7 (3.1)
	Mechanical–Chemical	64 (28.7)	67 (30.0)	80 (35.9)	211 (94.6)
	Biological–Chemical	1 (0.5)	3 (1.4)	1 (0.5)	5 (2.2)
Agricultural practices used in the field against FAW					
	Larvae killed by hand	56 (25.1)	51 (22.9)	62 (27.8)	169 (75.8)
	Egg mass destruction	20 (9.0)	32 (14.4)	38 (1.0)	90 (40.4)
	Tobacco	0	3 (1.4)	2 (0.9)	5 (2.2)
	Chilli powder	0	0	1 (0.5)	1 (0.5)
	Neem	0	0	0	0
	Ash	3 (1.4)	6 (2.7)	3 (1.4)	12 (5.4)
	Bishkatali	0	0	0	0
	Mud or sand	1 (0.5)	2 (0.9)	3 (1.4)	6 (2.7)
	Biocontrol agent	0	2 (0.9)	0	2 (0.9)
Used the chemicals in a season ***					
	1 time	3 (1.4)	3 (1.4)	0	6 (2.7)
	2 times	31 (13.9)	18 (8.1)	26 (11.7)	75 (33.6)
	3 times	18 (8.1)	38 (17.0)	50 (22.4)	106 (47.5)
	4 times	5 (2.2)	5 (2.2)	3 (1.4)	13 (5.8)
	>4 times	10 (4.5)	10 (4.5)	3 (1.4)	23 (10.3)
Intervals in use of chemicals ***					
	3 days	2 (0.9)	0	0	2 (0.9)
	7 days	33 (14.8)	51 (22.9)	36 (16.1)	120 (53.8)
	10 days	7 (3.1)	1 (0.5)	1 (0.5)	9 (4.0)
	15 days	22 (9.9)	19 (8.5)	45 (20.2)	86 (38.6)
Damage (%) in the absence of control measures of FAW					
	<10	0	2 (0.9)	1 (0.5)	3 (1.4)
	10–20	11 (4.9)	9 (4.0)	9 (4.0)	29 (13.0)
	21–30	6 (2.7)	12 (5.4)	20 (9.0)	38 (17.0)
	31–40	21 (9.4)	14 (6.3)	24 (10.8)	59 (26.5)
	>40	29 (13.0)	37 (16.6)	28 (12.6)	94 (42.2)

Numerals in parentheses are shown as percentage data. ^a^ Computed based on the number of farmer respondents concerning the experience of maize farming. *** and * denote significant differences between parameter and experience of maize farming at the 1 and 5% significance level, respectively, by Chi-square test.

**Table 4:** Pesticides used by farmers for fall armyworm control and their classification.

Pesticide Name (Trade Name with Formulation)	Active Ingredient	Freq. ^a^	%	WHO Class ^b^	DAE List ^c^	Applied in Stage of Plant
Nitro 505 EC	Chlorpyrifos (50%) + Cypermethrin (5%)	67	17.54	II	Y	Vegetative
Tracer 45 SC	Spinosad	60	15.71	III	Y	Vegetative
Karate 2.5 EC	Lambda Cyhalothrin	37	9.69	II	Y	Vegetative
Proclaim 5 SG	Emamectin Benzoate	36	9.42	II	Y	Vegetative
Success 2.5 SC	Spinosad	31	8.12	III	Y	Seedling, vegetative
Virtako 40 WG	Thiamethoxam (20%) + Chlorantraniliprole (20%)	31	8.12	Thiamethoxam—II Chlorantraniliprole—U	Y	Vegetative
Lumectin 10 WDG	Lufenuron (5%) + Emamectin Benzoate (5%)	18	4.71	II	Y	Vegetative
AC Mix 55 EC	Chlorpyrifos (50%) + Cypermethrin (5%)	13	3.40	II	Y	Seedling, vegetative
Saham 5 SG	Emamectin Benzoate	11	2.88	II	Y	Vegetative
Protect 5 SG	Emamectin Benzoate	10	2.62	II	Y	Vegetative
Setara 55 EC	Chlorpyrifos (50%) + Cypermethrin (5%)	9	2.36	II	Y	Vegetative
Sevin 85 SP	Carbaryl	9	2.36	II	Y	Vegetative
Morter 48 EC	Chlorpyriphos	8	2.09	II	Y	Vegetative
Master Plus 48 EC	Chlorpyriphos	8	2.09	II	Y	Vegetative
Reload 18 SC	Thiamethoxam (12%) + Fipronil (6%)	7	1.83	II	Y	Seedling, vegetative
Shobicron 425 EC	Profenofos (40%) + Cypermethrin (2.5%)	4	1.05	II	Y	Vegetative, flowering
Venom 25 WDG	Acetamiprid (20%) + Emamectin Benzoate (5%)	4	1.05	II	Y	Vegetative
Benten 1.8 EC	Abamectin	3	0.79	III	Y	Seedling, vegetative
Cartap 50 SP	Cartap	3	0.79	II	Y	Vegetative
Canopy 20 SL	Imidacloprid	3	0.79	II	Y	Flowering
Emitaf 20 SL	Imidacloprid	3	0.79	II	Y	Vegetative, flowering
Emithrin Plus 3% WD	Abamectin (1%) + Beta Cypermethrin (2%)	2	0.52	II	Y	Vegetative
Moxie 17.5 WDG	Imidacloprid (15%) + Lamda Cyhalothrin (5%)	2	0.52	II	Y	Vegetative, flowering
Catrapid 95 SP	Acetamiprid (3%) + Cartap (92%)	1	0.26	II	Y	Vegetative
Coragen 18.5 SC	Chlorantraniliprole	1	0.26	U	Y	Vegetative
Diazinon 60 EC	Diazinon	1	0.26	II	Y	Vegetative

^a^ Number of respondents (n = 223). ^b^ WHO recommended classification of pesticides by hazard and guidelines to classification. ^c^ Pesticide list obtained from Department of Agricultural Extension (DAE).

## Data Availability

The datasets generated and analyzed during the current study are available from the corresponding author on reasonable request.

## Supplementary Materials

The following supporting information can be downloaded at: https://www.mdpi.com/article/10.3390/insects14040343/s1, File S1: Informed Consent and Questionnaire.
