# Supplementary material for: Invasion, Distribution, Monitoring and Farmers Perception of Fall Armyworm (Spodoptera frugiperda) and Farm-Level Management Practices in Bangladesh
Source: Insects. 2023 Mar 31;14(4):343. doi: 10.3390/insects14040343 (PMC10143613; doi:10.3390/insects14040343)
Supplement: Supplementary file 1 [file insects-14-00343-s001.zip › insects-2246100-supplementary.pdf]

File S1: Informed Consent and Questionnaire

INFORMED CONSENT (BLANK FORM)

The aim of this survey is collecting on farmers' pest management decisions based on their knowledge, perceptions, and practices and focused on: (1) basic information of the interviewee, such as gender and cultivable land owned; (2) practices for maize production, such as varieties grown and experience; (3) experience with FAW infestation, such as damage season and month of highest infestation; and (4) farmer's knowledge, perceptions, and practices concerning FAW management.

The study is promoted by the Department of Entomology, Bangladesh Agricultural University, Mymensingh. The questionnaire is completely anonymous, and data will be collected and managed in aggregated form according to Bangladesh Agricultural University System (BAURES).

Do you agree to participate to the study?

[ ] I agree [ ] I do not agree

Questionnaire on Fall Armyworm in Bangladesh

Date of Interview:

Name of Farmer:

Address:

Village:..................................Upzilla:.............................Zilla:..

1. Experience in Maize Cultivation: .... years

2. Maize Cultivation Land: .... Acres

3. Corn varieties:

4. At what time is fruit armyworm more common? (mark √)
    a. Rabi (Winter) – More/ Less
    b. Kharif 1 (early summer) – more/less
    c. Kharif 2 (end of summer) – more/less

5. At which stage of the crop are fruit armyworms more common? (mark √)
    a. Seedling stage
    b. Vegetative stage
    c. Reproduction or flowering stage
    d. Fruiting stage

6. In which month is fruit armyworm attack more common? (mark √)
    a. January
    b. February
    c. March
    d. April
    e. May
    f. June
    g. July
    h. August
    i. September
    j. October
    k. November
    l. December

7. How do you control the fruit armyworm in the field? (mark √)
    a. Physical means
    b. Mechanical means
    c. Biocontrol means
    d. Chemical means
    e. By local means (.................................)

8. How do you control fruit armyworms locally? (mark √)
    a. Killing worms with hands
    b. Destroys the egg pile
    c. Gul or tobacco leaves
    d. Chilli powder solution
    e. Neem medicine
    f. Ash
    g. Bishkatali
    h. muddy water
    i. Biocontrol agents Other (............................)

9. Do you recognize these biological agents? (mark √)
    a. *Bracon*
    b. *Trichogramma*
    c. *Bacillus thuringiensis*

d. SfNPV
e. Lady Bird Beetle
f. Spider
g. Fortenza
h. Foulizen
i. Other (............................

10. What chemicals do you use to control fruit armyworm?

	Chemical Name	Dose (ml/g per acre)	crop stage
1			
2			
3			
4			
5			

11. How often do you use chemicals to control fruit armyworms? (mark √)
   a. Once
   b. Twice
   c. Three times
   d. Four times
   e. More than four times

12. How often do you use chemicals to control fruit armyworm? (mark √)
   a. Every 3 days
   b. Every 7 days
   c. Every 15 days
   d. Other (.........)

13. What percentage of corn is damaged if fruit armyworm is not controlled? (mark √)
   a. More than 10%
   b. 11-20%
   c. 21-30%
   d. 31-40%
   e. More than 40%

Name of data collector:
